# Social undermining and psychological empowerment: unveiling the association to resilience in nursing - a cross-sectional study

**DOI:** 10.1186/s12912-025-03260-0

**Published:** 2025-06-17

**Authors:** Heba Abdelfatah Ahmed, Abdelaziz Hendy, Ahmed Abdellah Othman, Ahmed Gamaleldin Mohamed Attia, Nadia Mohamed Ibrahim Wahba

**Affiliations:** 1 Psychiatric Nursing and Mental Health, Faculty of Nursing, Damietta University, Damietta City, Egypt; 2 Department of Pediatric Nursing, Faculty of Nursing, Ain Shams University, Benha City, Egypt; 3 Department of Nursing Administration, Faculty of Nursing, Sohag University, Sohag City, Egypt; 4 Psychiatric and Mental Health Nursing, Faculty of Nursing, Cairo University, Giza City, Egypt; 5 Department of Nursing, North Private College of Nursing, Arar, Saudi Arabia; 6 Psychiatric Nursing and Mental Health, Faculty of Nursing, Port Said University, Port Said City, Egypt; 7 College of Nursing, Prince Sattam Bin Abdulaziz University, Al-Kharj, 11942 Saudi Arabia

**Keywords:** Nursing, Resilience, Psychological empowerment & social undermining

## Abstract

**Objective:**

This study intended to explore the mediating effect of psychological resilience in the relationship between social undermining and psychological empowerment among nurses.

**Methods:**

A cross-sectional correlational descriptive design was employed to fulfill the study’s aim at Damietta General Hospital through convenient sample of 385 registered nurses. Data analysis was implemented using SPSS software package version 26.0.

**Findings:**

Study findings reported that a direct effect of social undermining on psychological resilience is negative and significant (β = -0.092, SE = 0.042, Z = -2.17, *p* = 0.030). Similarly, psychological resilience significantly predicts psychological empowerment with a strong positive effect (β = 0.347, SE = 0.047, Z = 7.26, p = < 0.001). The direct effect of social undermining on psychological empowerment is negative and highly significant (β = -0.422, SE = 0.036, Z = -11.51, p = < 0.001).

**Conclusion:**

The study concluded that social undermining is a workplace stressor that emotionally damages employees by making them feel burnt out when performing their job. It also has a negative influence on psychological empowerment of employees, and in reducing their psychological resilience. Also, psychological resilience mediates social undermining and psychological empowerment.

**Nursing implications:**

It is crucial for organizations to seek for promoting ethical leaders through creating an ethical infrastructure, which can forestall social undermining. Emphasizing the implementation of interventions aimed at building resilience as a strategy for mitigating the harmful effects of social undermining. Overall, this research underscores the interconnectedness of social undermining and psychological empowerment, emphasizing the necessity for supportive organizational environments that promote both resilience and psychological empowerment.

## Introduction

Nurses are the principal healthcare providers in hospitals and are responsible for providing the majority of long-term care [1]. As a fact, nurses often work in stressful settings where they must provide compassionate care under tough circumstances [2]. To guarantee the quality and sustainability of care and foster safety of nurses, health settings need to address main factors, which jeopardize performance of health team [3].

Unsurprisingly, social undermining is one of the critical workplace issues confront nurses in the health settings [4, 5]. Scholars define social undermining as a kind of mistreatment characterized by “perception of others’ expressions of negative affect (e.g., anger), negative evaluation, and behavior which hinder goal attainment” [6]. These behaviors include competing for positions, providing misleading information, delaying colleagues’ work to slow them down, devaluing colleagues’ ideas, and spreading false rumors about colleagues. Social undermining can also be indirect, taking the form of offensive comments about colleagues or ignoring them [7, 8].

Increased sense of being a target of undermining behaviors had a harmful impact on mental health, causing symptoms such as distress, anxiety, despair, and burnout [9]. Moreover, undermining can cause struggle among nurses and affect hospital morale, productivity and professional relations [10]. Consequently, nurses exposed to workplace social undermining feel uncomfortable and do not trust to share information with colleagues as they have negative expectations of how colleagues will utilize this information [11].

In Egyptian healthcare settings, majority of nurses are experienced high and moderate levels of undermining by supervisors and colleagues (such as workplace bullying, gossip, and exclusion) as nurses confront many stressors resulting from dealing with severely ill patients, shortage of staff and scarcity of resources. Thus, they are more likely to engage in workplace undermining behaviors that had negative impact on their health and career [4]. According to Yu et al.’s study, resilience aid in reducing negative impact of undermining [6].

Resilience among Egyptian nurses has been the focus of several recent studies, especially in time of adversity as COVID-19 [12]. In nursing, resilience defined as the degree of a nurse’s capability to deal with workplace stressors and mental health pressures. Resilient individuals are emotionally quieter when dealing with unacceptable situations [13]. In nursing, resilience has been found to be a protective factor against adversity. In times of crisis, resilience enables nurses to maintain their mental and emotional health [14–16].

The advantages of resilience that are most frequently mentioned are averting negative psychological consequences, demonstrating good coping and adaptation techniques, enhancing self- and other- awareness, and delivering superior nursing care [17]. Resilience in the nursing profession can improve client and staff outcomes, as well as care quality. Besides, it brings job success which in turn inspire nurses’ positive proactive behaviors, resilience as well as decreased turnover intention in the workplace [18, 19]. Resilient nurses are characterized by intelligence, self-assurance, ingenuity, flexibility, positivity, passion, good problem-solving abilities, and strong communication skills [20, 21].

Concerning the relationship between resilience and social undermining, there is a negative relation. The psychological effects of social undermining, as loneliness and low self-esteem, have a direct influence on nurses’ resilience [22]. Social undermining compromises a nurse’s resilience by impairing not just their mental health but also their ability to accomplish their work. Employees with a high level of resilience could better struggle negative impacts of perceived social undermining [6].

With the immense responsibilities of the nursing profession, psychological empowerment (PE) has a great importance. By definition, psychological empowerment is a cognitive and attitudinal mental status that helps nurses to feel competent in carrying out their designated activities [23]. It is the best and effective way to enhance productivity, make use of the available organization’s resources, and release potential skills in order to accomplish the objectives. It is often known that psychological empowerment and nurses’ job satisfaction are associated [24].

Psychological empowerment in healthcare services is pivotal for promoting the quality of patient care [25]. When health professionals, especially nurses, are continuously empowered, they experience increasing in autonomy, leading to improvement in patient conditions, enhanced communication, higher job satisfaction, and retention [26, 27]. Empowered nurses are more motivated to take responsibility, make right decisions, be assertive, innovative and supporter for their care recipients [28]. Additionally, empowerment promotes innovation, continuous development, and a patient-focus approach, ultimately leading to a more effective and compassionate healthcare delivery system [29].

Regarding the interplay between resilience and psychological empowerment, PE strengthens resilience by promoting a sense of control, advancing professional development, and fostering effective commitment and performance [30]. Previous scholars revealed that resilience has positive effect on PE by enhancing positive attitudes that help individuals to feel competent and in control of their career. Resilient individuals are more likely to recognize their duties as impactful and meaningful that are critical components of psychological empowerment [31]. In nursing field, empowered nurses have the self-assurance and tools necessary to handle any obstacles at work so that they demonstrate better levels of resilience [32].

With reference to the interplay between resilience and psychological empowerment and undermining, resilience enhances self-efficacy, self-esteem and internal motivation of employees, which considered key dimensions of psychological empowerment. empowerment has also found to alter the relationship between resilience and hardship at work. For instance, Ren and kim’s study [33] found that nurses who experience psychological empowerment are more robust to the adverse impacts of social undermining.

Additionally, empowerment alleviates the stress and unhappiness associated with undermining behaviors, allowing nurses to remain resilient and maintain their high performance standards. A study by Tello [34], concluded that the meaning and competence components of psychological empowerment are most strongly associated with resilience. furthermore, psychological empowerment is critical in helping nurses retain resilience, especially in high-pressure circumstances such as intensive care units [35] so that nurses with high levels of empowerment demonstrated strong resilience, coping better with job stress and work challenges. Social undermining, psychological empowerment, and resilience have a complicated and nuanced interaction. Therefore, this study was conducted to disclose the relationship between psychological empowerment and social undermining in relation with resilience among nurses in Egypt.

## Aim of the study

This study intended to explore the mediating effect of psychological resilience in the relationship between social undermining and psychological empowerment among nurses.

### The study hypotheses were as follows

#### Hypothesis 1

Social undermining has a negative effect on psychological empowerment among nurses.

#### Hypothesis 2

Psychological resilience and psychological empowerment among nurses are positively correlated.

#### Hypothesis 3

Social undermining has a negative impact on psychological resilience among nurses.

#### Hypothesis 4

Psychological resilience mediates the relationship between social undermining and psychological empowerment among nurses.

**Fig. 1:**
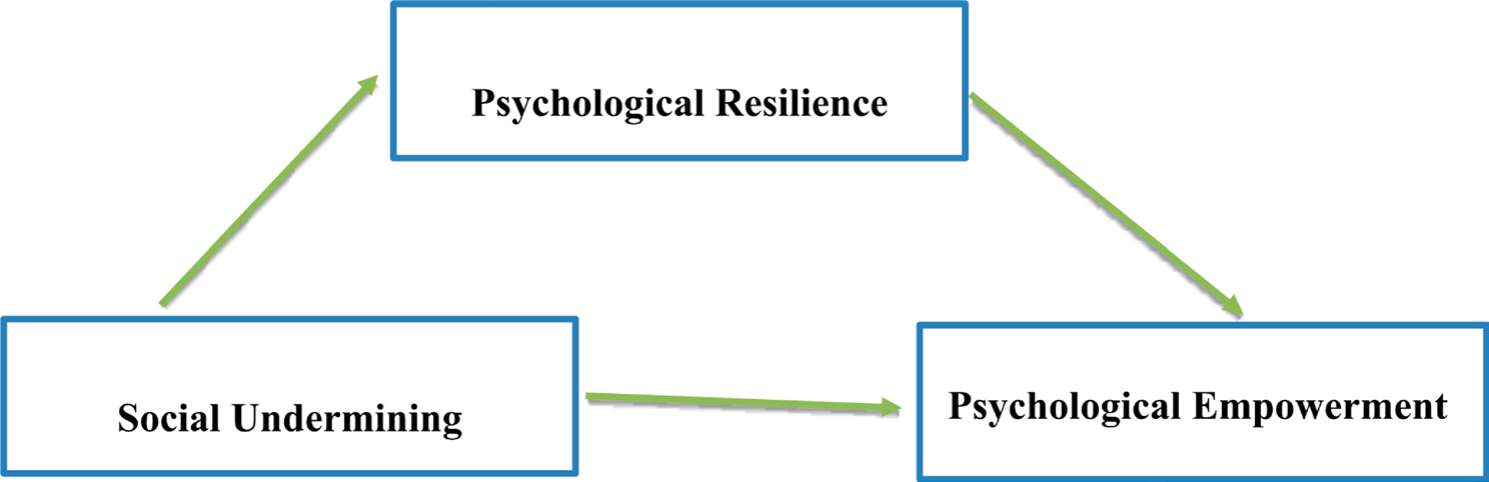
Conceptual framework developed for this study

The conceptual framework of the current study was created using the preceding hypotheses as a guide, as illustrated in Fig. 1.

## Subjects and method

### Study design and setting

A cross-sectional correlational descriptive design was employed to fulfill the study’s aim. This study was conducted at Damietta General Hospital. It is a major qualitative leap in the health system in Damietta Governorate. This hospital has top-notch medical and health departments that provide patients with effective care in a variety of specialties. Emergency, medical, surgical, intensive care, renal dialysis, obstetric, pediatric, operating theater, radiology, and outpatient are among these departments. It accommodates about 500 beds. It serves large residents with a broad catchment area.

### Study participants

The study participants encompassed a convenient sample of registered nurses recruited from diverse departments within the aforementioned setting. The sample size was calculated utilizing the subsequent formula: **(n) =** (Z^2 * P * (1 - P)) / E^2, **Where: Z =** Z-value (the number of standard deviations from the mean that corresponds to the desired confidence level, usually 1.96 for a 95% confidence level). **P =** Estimated prevalence or proportion of the population with the characteristic being studied (if unknown, we often use 0.5 for maximum variability). **E =** Margin of error (precision), the maximum acceptable difference between the sample statistic and the population parameter (usually 0.05 for a 5% margin of error). Using the assumptions: **Z =** 1.96 (for 95% confidence), **P =** 0.5 (maximum variability), **E =** 0.05 (desired precision), **n =** (1.96^2 * 0.5 * (1–0.5)) / (0.05^2) = 384.16, So, the required sample size would be approximately **385** participants.

Recruitment criteria for the staff nurses included in the study comprising the succeeding: Must have at least 6 months of experience in the department in which they presently work, full-time employment, putting in at least 20 h per week, from both sexes, and expressing a desire and willingness to take part in the study.

### Data collection instruments

Data for the current study was collected using the subsequent four instruments, which included:

### Nurses’ personal and job characteristics data sheet

This structured sheet was developed by the researchers, it comprised personal and job characteristics encompassing nurses’ age, sex, marital status, academic qualifications, and years of experience, as well as working department.

### Connor and davidson resilience scale (CD-RISC-25)

This scale was created by Connor and Davidson [36] to measure an individual’s ability to adjust to challenging life situations, preserve emotional stability and navigate and rebound from hindrances and after exposure to adversity and traumatic events. The CD-RISC encompasses 25 items. The five dimensions that make up the CD-RISC-25 represent various facets of resilience, including personal competence (8 items), trust in one’s instincts (7 items), positive acceptance and secure relationships (5 items), control (3 items), and ultimately spiritual influences (2 items). Each item is rated by the participants on a 5-point Likert scale from 0 (not true at all) to 4 (true nearly all the time), with an entire score spanning a range of 0-100 points. Higher scores on the CD-RISC-25 reveal higher levels of psychological resilience. Signifying worthy validity and reliability of the CD-RISC- 25, the results of a Jordanian study conducted by Alfuqaha [37] indicated by a total Cronbach’s alpha (α) value of 0.93, with 0.74, 0.77, 0.88, 74, and 0.82 for positive acceptance and secure relationships, trust in one’s instincts, personal competence, control, and spiritual influences respectively.

### Psychological empowerment scale (PsyES)

The Spreitzer’s Psychological Empowerment Scale developed by Spreitzer [38]. It is a self-report questionnaire that takes into account people’s possible thoughts about their work role. The twelve items on this scale are evenly split among the four dimensions of competence or self-efficacy, meaning, impact, and self-determination. Separately item was ranked by respondents using a seven-point Likert scale ranging from 1 (= Strongly disagree) to 7 (= Strongly agree), and there is no converse scored items. Validity estimates for the dimensions are typically around 0.80, and test-retest method affirmed its robust reliability [38].

### Social undermining scale (SUS-26)

Duffy et al. [39] devised the social undermining scale as a robust tool takes into account assessing individuals’ perceptions of both supervisor and coworker social undermining behavior. It encompasses 26 items (13 items each), utilizing a 6-point Likert scale ranging from 1 (Not at all) to 6 (Everyday) for participant responses. Strong psychometric properties and stability of the SUS-26 were affirmed, demonstrating creditable reliability, the scale yielded Cronbach’s α values of a total of 0.80, with 0.92 for supervisor undermining and 0.90 for co-worker correspondingly [39]. In this respect, the scale demonstrated vigorous reliability with values of 0.93 and 0.94 for supervisor and co-worker social undermining correspondingly [5].

### Instruments’ validity and reliability

A panel of knowledgeable nursing professionals from the departments of Psychiatric Nursing and Mental Health, and Nursing Administration critically reviewed all the study instruments in order to assess its face and content validity. They provided feedback on the questionnaires’ overall scope, usability, and applicability. Their invaluable input resulted in specific modifications, such as the elucidation of potentially perplexing elements and the modification of certain terms to more accurately represent local cultural contexts.

To establish the reliability and trustworthiness of the Social Undermining Scale, the Cronbach’s alpha coefficient test was computed, yielding results of values of a 0.86 overall, with 0.92 for supervisor undermining and 0.84 for co-worker respectively. Regarding Connor and Davidson Resilience Scale, it has demonstrated a satisfactory internal consistency with Cronbach’s alpha values of 0.90 for overall scale, and 0.85, 0.79, 0.76, 0.80, and 0.84 for its five dimension comprising personal competence, trust in one’s instincts, positive acceptance and secure relationships, control, and finally spiritual influences correspondingly. As well as, Psychological Empowerment Scale affirmed its robust reliability with Cronbach’s alpha values of 0.86.

### Pilot study

Pilot research was conducted on 10% of all the registered nurses being surveyed (*n* = 39) who were selected at random. The pilot study evaluated the time needed to complete the instruments, tested their clarity, applicability, and feasibility, and served as a final check for comprehensibility and cultural suitability, as well as identifying any obstacles or issues that might prevent the process of gathering data. No changes were made based on the pilot study’s findings as the study instruments were clear and easily understood.

### Data collection process

Data for the current study was collected utilizing self-administered handwriting questionnaire over the course of four months from September 2024 to December 2024. After properly illuminating the study’s inducement, an official permission from the aforementioned setting’s authorities was obtained to confirm their cooperation and guarantee to conduct the study. To gain registered nurses’ plentiful collaboration, the study’s objectives, methods, and their right to withdraw from the study at any time without any repercussions were all thoroughly explained during the individual meetings that were used to recruit participants. Before beginning to fill out the questionnaire, each registered nurse who met the eligibility criteria gave his\her informed written consent. Participants were told to submit their completed questionnaire anonymously in order to conserve confidentiality. In an effort to obtain a variety of viewpoints, the researchers prudently distributed the questionnaire to registered nurses with guarantying coverage across various shifts and working hours. The researchers purposefully tried to collect the data at the conclusion of the nurses’ shifts in order to prevent participants from giving hurried and thoughtless answers.

The researchers supposed that by giving registered nurses enough time to focus and finish the questionnaire, the competence and dependability of the information gathered would be improved. Participants took an estimated 15 to 20 min to finish the questionnaire. The researchers carefully examined every questionnaire they received in order to guarantee the integrity and accuracy of the data they had gathered. This thorough review procedure was started right away to address any possible missing, inconsistent, or omitted data. The registered nurses were then acknowledged for their generous donation of their time and effort.

### Ethical considerations

An ethical approval to carry out this study was secured from the Scientific Research Ethics Committee at Damietta University, Egypt, the Subcommittee for the Medical Sector, Faculty of Nursing, identified by a reference number (Du Rec no 32 on Aug 20, 2024). Study performed in accordance with the principles of Declaration of Helsinki with experiments on humans and/or the use of human tissue. Informed consent to participate was obtained from all of the participants in the study. The privacy and anonymity of the participants were rigorously preserved. Besides that, the freedom to decline study participation or to withdraw from the study at any time without facing any unfavorable consequences was guaranteed.

### Statistical analysis

Initially, a computer was utilized for data entry. Subsequently, meticulous review was done after the data was entered to avoid any mistakes during data input. Data analysis was implemented using SPSS software package version 26.0 (IBM Inc., Chicago, IL, USA). The Cronbach’s alpha coefficient was used to measure the internal consistency of the study’s instruments. The participants’ personal and job characteristics were described using descriptive statistics including numbers and percentages. Study variables were presented utilizing means and standard deviations (SD). The correlation matrix among the study’s quantitative variables was pronounced with Pearson’s correlation analysis. To determine whether social undermining and psychological resilience predict psychological empowerment, regression analyses were employed to determine the direct effect of social undermining on psychological empowerment. A structural equation model was used to examine the indirect effect of social undermining on psychological empowerment among registered nurses through psychological resilience. The attained findings were considered significant if *p* < 0.05.

The model of Structural Equation Modeling adequacy was evaluated using multiple fit indices, including the Chi-square statistic (χ²), Root Mean Square Error of Approximation (RMSEA), Comparative Fit Index (CFI), Tucker-Lewis Index (TLI), and Standardized Root Mean Square Residual (SRMR). The results indicated a good model fit: χ²(98) = 145.32, *p* < 0.001; RMSEA = 0.045; CFI = 0.96; TLI = 0.95; and SRMR = 0.042.

## Results

Table 1. the demographic profile provides a detailed breakdown of the 385 registered nurses’ characteristics in terms of age, sex, marital status, educational level, working department, and years of experience. The majority of participants fall within the 30 **- <**40-year- old group (48.8%), with a mean age of 29.8 years (SD = 5.4). The sample comprises a higher proportion of females (57.9%). Marital status shows diversity, with most participants are married (70.9%). In terms of educational level, a significant portion holds a bachelor’s degree (67.5%), followed by technical education (19%). Regarding working departments, the majority are employed in intensive care (39.5%). The experiences levels are spread across diverse categories, with a significant proportion of participants have 5 to 10 years of experience (40.3%). The mean experience level is 7.5 years (SD = 5.02).

**Table 1:** Characteristics of studied nurses (*n* = 385)

Characteristics	N	%
Age: 20 - < 30 30 - < 40 40 - ≥ 50	159 188 38	41.3 48.8 9.9
Mean (SD)	29.8 (5.4)	
Sex: Male Female	162 223	42.1 57.9
Marital status: Single Married Divorced Widow	100 273 3 9	25.9 70.9 0.8 2.4
Education level: Technical education Bachelor’s degree Postgraduate degree	73 260 52	19 67.5 13.5
Working department: Intensive care Emergency Medical Dialysis Pediatric Operation Surgical Outpatient Obstetric	152 91 34 28 27 22 13 12 6	39.5 23.6 8.8 7.3 7 5.7 3.4 3.1 2.6
Years of experience: < 5 5–10 > 10	107 155 123	27.8 40.3 31.9
Mean (SD)	7.5 (5.02)	

SD: Standard Deviation

Table 2 Shows that the dimensions of social undermining, supervisor social undermining had a mean of 28.68 (SD = 11.7), with values ranging from 13 to 72, while coworker social undermining showed a similar mean of 27.41 (SD = 10.14), ranging from 13 to 53. Total social undermining had the highest variability, with a mean of 56.09 (SD = 20.39) and values spanning from 29 to 119. In terms of psychological outcomes, psychological empowerment had a mean of 55.11 (SD = 16.9), ranging from 34 to 84, indicating moderate psychological empowerment level. Psychological resilience demonstrated a lower mean of 39.17 (SD = 17.01), ranging from 21 to 88, reflecting variability in resilience levels among participants.

**Table 2:** Descriptive statistics of study variables (Mean ± SD and Range) (*n* = 385)

Study variables	Mean ± SD	Max- Min
Supervisor Social Undermining	28.68 ± 11.7	72 − 13
Coworker Social Undermining	27.41 ± 10.14	53 − 13
Total Social Undermining	56.09 ± 20.39	119 − 29
Psychological Empowerment	55.11 ± 16.96	84 − 34
Psychological Resilience	39.17 ± 17.01	88 − 21

SD: Standard Deviation

**Fig. 2:**
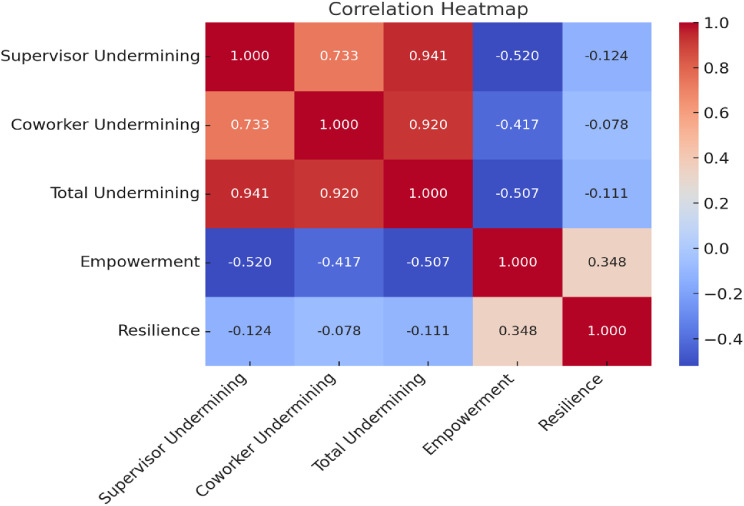
Correlation Heatmap among the study variables (*n* = 385)

Table 3 and Fig. 2. reveal that supervisor social undermining exhibited a strong positive correlation with coworker social undermining (*r* = 0.733, *p* < 0.001) and total social undermining (*r* = 0.941, *p* < 0.001), indicating their interconnected nature. Conversely, supervisor social undermining demonstrated a moderate negative correlation with psychological empowerment (*r* = -0.520, *p* < 0.001) and a weak negative correlation with psychological resilience (*r* = -0.124, *p* < 0.05). Similarly, coworker social undermining was strongly correlated with total social undermining (*r* = 0.920, *p* < 0.001) and negatively associated with psychological empowerment (*r* = -0.417, *p* < 0.001), but it showed no significant relationship with resilience (*r* = -0.078, *p* = 0.125). Total social undermining was moderately negatively correlated with psychological empowerment (*r* = -0.507, *p* < 0.001) and weakly negatively correlated with resilience (*r* = -0.111, *p* < 0.05). Finally, psychological empowerment and resilience demonstrated a weak positive relationship (*r* = 0.348, *p* < 0.001).

**Table 3:** Correlation matrix among the study variables (*n* = 385)

Study variables		Supervisor social undermining	Coworker social undermining	Total social undermining	Psychological empowerment	Psychological resilience
Supervisor Social Undermining	*r* *p*		0.733 > 0.001	0.941 > 0.001	− 0.520 > 0.001	− 0.124 0.015
Coworker Social Undermining	*r* *p*			0.920 > 0.001	− 0.417 > 0.001	− 0.078 0.125
Total Social Undermining	*r* *p*				− 0.507 > 0.001	− 0.111 0.030
Psychological Empowerment	*r* *p*					0.348 > 0.001
Psychological Resilience	*r* *p*					

*r* Pearson coefficient *p* p.value

**Fig. 3:**
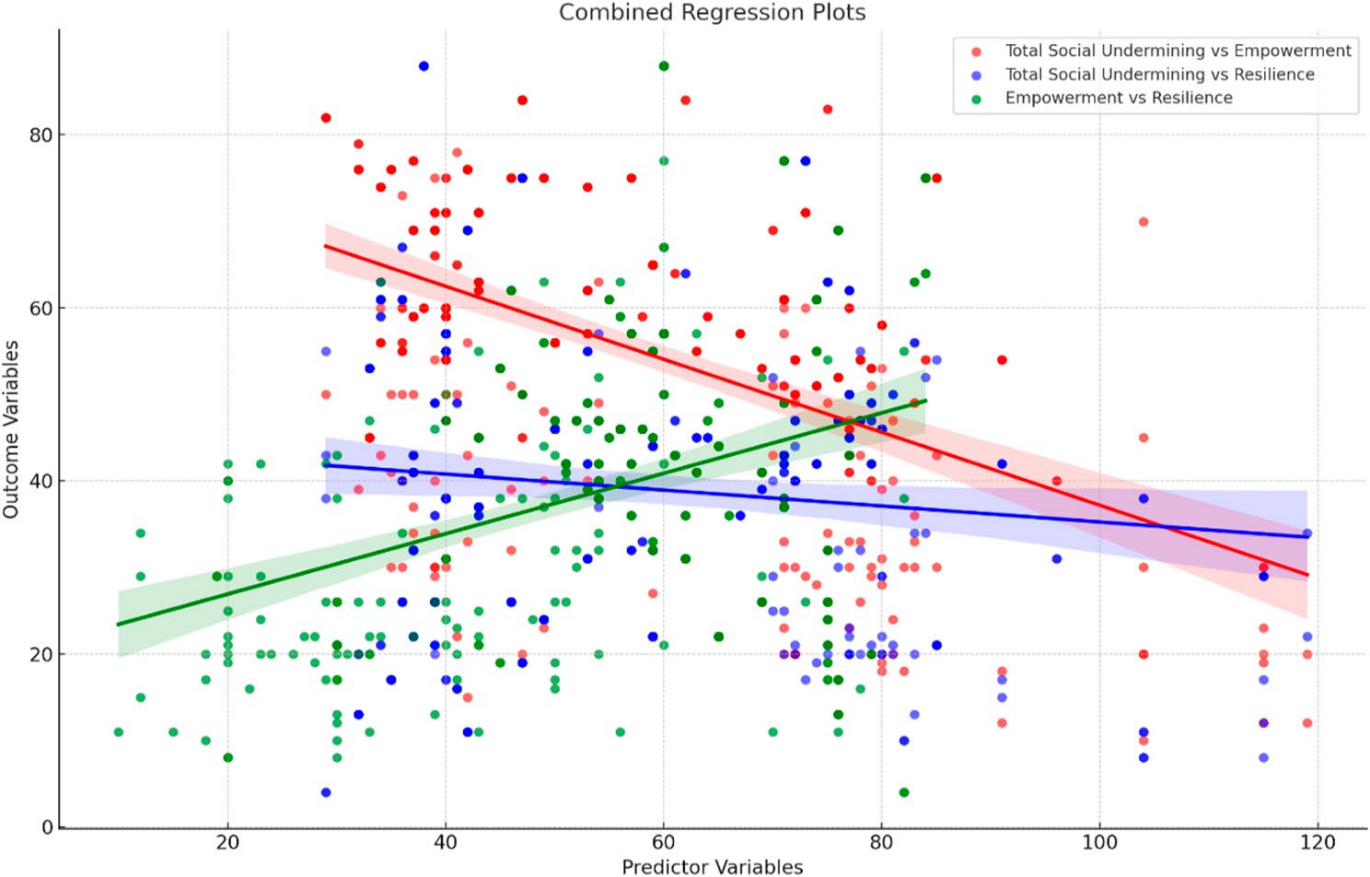
Relationships among total social undermining, psychological empowerment, and psychological resilience among the studied nurses (*n* = 385)

Figure 3 illustrates that there is a negative relationship between total social undermining and psychological empowerment. The trend line shows a moderate negative slope, indicating that undermining environments are associated with reduced empowerment levels. Higher levels of total social undermining slightly reduce psychological resilience, as shown by the shallow downward trend. Lastly, there is a positive relationship between psychological empowerment and psychological resilience. The trend line shows a positive slope, suggesting that empowerment enhances resilience levels, even in challenging environments.

**Fig. 4:**
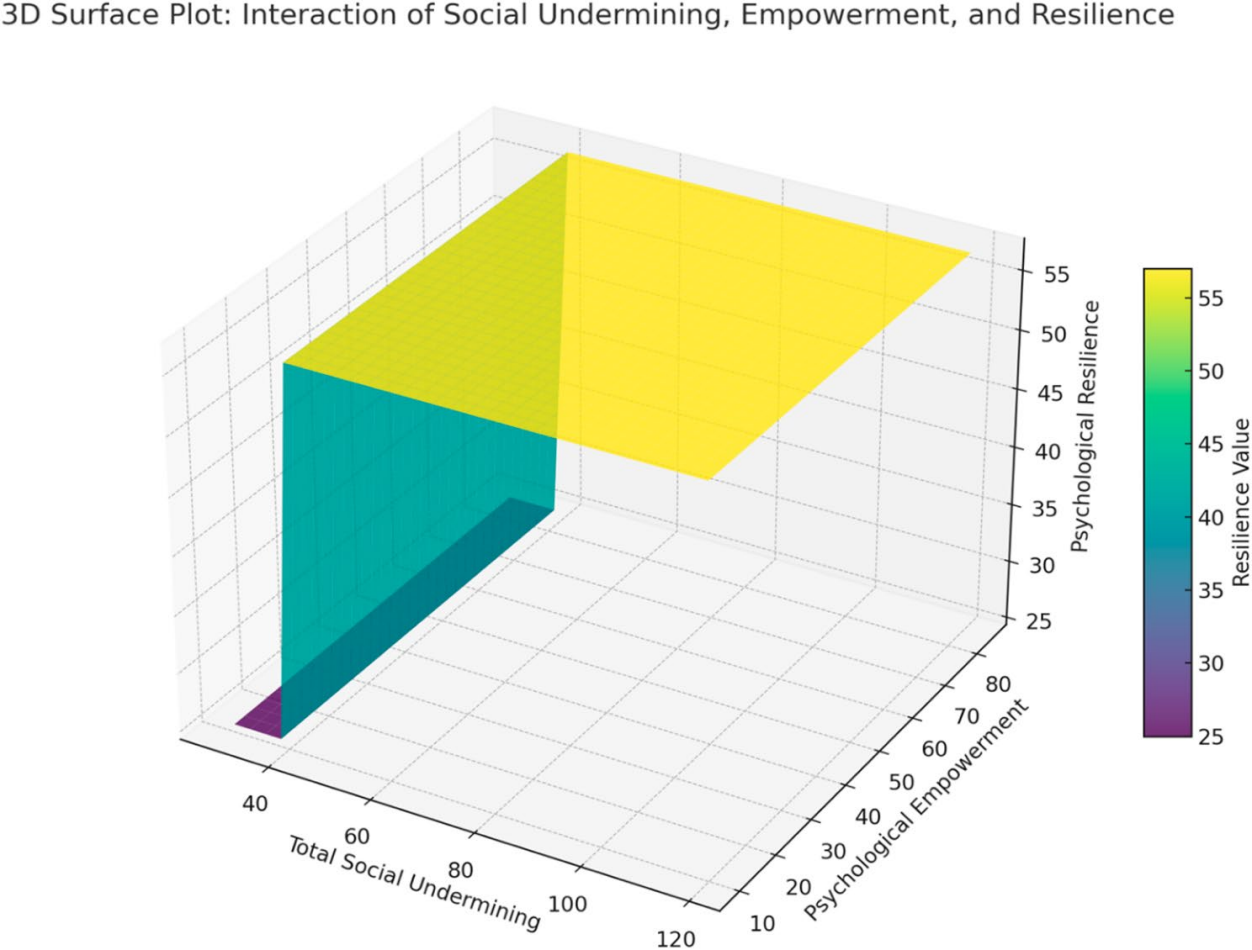
The 3D surface plot visualizes the interaction between total social undermining, psychological empowerment, and psychological resilience

Figure 4 as is evident, the surface plot helps visualize the combined effect of total social undermining and psychological empowerment on psychological resilience. As total social undermining increases, it seems to generally have a negative impact on psychological resilience. Psychological empowerment appears to counterbalance this effect; as higher empowerment values correspond to higher resilience despite increasing social undermining.

**Fig. 5:**
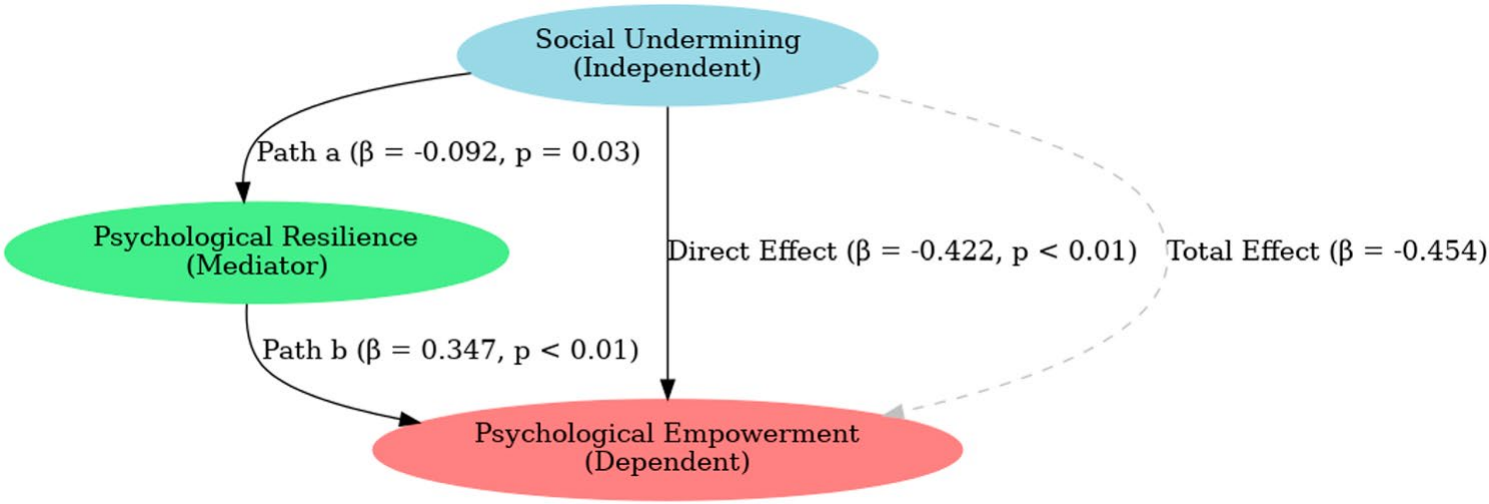
Structural equation model with social undermining as the independent variable, psychological resilience as the mediator, and psychological empowerment as the dependent variable

Table 4 and Fig. 5 further demonstrate the mediation analysis which reveals several significant relationships among the variables. The direct effect of social undermining on psychological resilience is negative and significant (β = -0.092, SE = 0.042, Z = -2.17, *p* = 0.030*), suggesting that higher levels of social undermining reduce resilience. Similarly, psychological resilience significantly predicts psychological empowerment with a strong positive effect (β = 0.347, SE = 0.047, Z = 7.26, p = < 0.001), indicating that resilience enhances empowerment. The direct effect of social undermining on psychological empowerment is negative and highly significant (β = -0.422, SE = 0.036, Z = -11.51, p = < 0.001), showing that social undermining strongly diminishes empowerment.

**Table 4:** Mediation effect of psychological resilience between social undermining and psychological empowerment among the studied registered nurses (*n* = 385)

Study variables	β	SE	Z. value	*p*. value
Direct (Social undermining → Psychological resilience)	-0.092	0.042	-2.17	0.030
Direct (Psychological resilience → Psychological empowerment)	0.347	0.047	7.26	> 0.001
Direct (Social undermining → Psychological empowerment)	-0.422	0.036	-11.51	> 0.001
Path a: Relationship between Social undermining → Psychological resilience	-0.092	0.042	-2.17	0.030
Path b: Psychological resilience → Psychological empowerment	0.347	0.047	7.26	> 0.001
Indirect Effect (Social undermining → Psychological empowerment via Psychological resilience)	-0.032	0.044	-0.71	> 0.001
Total Effect (Social undermining → Psychological empowerment)	-0.454	0.058	-7.82	> 0.001

β: Beta, standardized regression coefficient SE: Standard error

Path a (social undermining → psychological resilience) and path b (psychological resilience → psychological empowerment) reinforce these findings, with β values of -0.092 and 0.347, respectively. As well as, by employing a structural equation model, the indirect effect of social undermining as an independent variable on psychological empowerment as an outcome variable via psychological resilience as a mediating variable is small but significant (β = -0.032, SE = 0.044, Z = -0.71, p = < 0.001). Finally, the total effect of social undermining on psychological empowerment (direct + indirect) is negative and highly significant (β = -0.454, SE = 0.058, Z = -7.82, p = < 0.001), emphasizing the overall detrimental impact of social undermining on empowerment. These results highlight the importance of resilience as a mediator in mitigating the negative effects of social undermining on empowerment.

## Discussion

Social undermining is indeed a significant concern within the nursing profession, characterized by behaviors aimed at obstructing colleagues’ efforts to foster positive relationships and maintain their professional integrity. This behavior can arise from both supervisors and coworkers, creating a toxic work environment that jeopardizes team cohesion. The adverse consequences of social undermining are far-reaching, negatively impacting nurses’ self-confidence, interpersonal relationships, and communication quality with supervisors, coworkers and patients. These effects are especially critical in high-stress environments like critical care settings, where the demands of complexity and time constraints exacerbate the challenges faced by nursing staff, ultimately threatening the quality of patient care [4]. Therefore, the present study’s focus on examining the mediating role of psychological resilience in the relationship between social undermining and psychological empowerment among nurses.

The findings regarding social undermining among nurses are noteworthy, particularly given that over half of the participants reported high levels of total social undermining. Specifically, the mean score for coworker social undermining was slightly lower than a mean score of supervisor social undermining, indicating that some nurses experience significant challenges due to undermining behaviors from both supervisors and coworkers. This prevalence may be attributed to the high representation of nurses working in high-pressure departments like intensive care units and emergency departments, which inherently involve significant stressors such as managing critically ill patients, staff shortages, and resource limitations. Such conditions can contribute to heightened emotional strain, making nurses more vulnerable to experiencing undermining behaviors.

This result in is a parallel with findings of Obied et al., and Jung & Yoon [4, 40] who revealed similar perceptions of high levels of workplace social undermining among nurses. In contrast, Lyu et al. [41], conveyed an average score for perceived abusive supervision suggesting that abusive supervision may not be prevalent among nurses in their study. Overall, the dynamics of workplace relationships, particularly in stressful settings, can significantly influence nurses’ psychological outcomes, including their sense of empowerment and job satisfaction.

The current study’s results revealed that, the mean score of psychological empowerment among nurses indicating a moderate level with significant variability. This variability relates to the moderate levels of social undermining experienced by nurses, particularly from supervisors (28.61) and coworkers (27.84). High perceived social undermining can erode confidence and reduce a nurse’s sense of agency, resulting in lower empowerment scores. Stressful environments, like intensive care units (ICUs) and emergency departments (ERs), further exacerbate feelings of inadequacy. Support systems are crucial; nurses with strong support report higher empowerment, while those facing social undermining often feel isolated or undervalued.

Additionally, the relationship between resilience and empowerment is vital, as resilient nurses can maintain empowerment despite challenges, whereas those with lower resilience may experience heightened negative effects. Overall, these factors underscore the need for supportive interventions to combat social undermining and enhance resilience, ultimately improving nurses’ psychological empowerment and job satisfaction.

Analogous with the preceding findings, Lyu et al. [41], who conducted a survey to identify “the mediating effects of nurses’ psychological empowerment on abusive supervision and turnover intention as perceived by nurses,”

clarified an average psychological empowerment score among clinical nurses, indicating a moderate-to-high level of empowerment. On the contrary, Allowh et al.’s study [42] revealed that nurses perceived a high level of empowerment within their working units. Additionally, the current results are resonated with Khrais and Nashwan [43] who reported mild to moderate levels of psychological empowerment.

The mean score of psychological resilience among nurses in the existing study indicating a moderate level, which may be influenced by various demographic factors. For instance, higher proportion of the nurses are under 30 years old, they may struggle more with workplace stress, resulting in lower resilience scores compared to older colleagues. Additionally, most of nurses work in high-stress units like the ICUs and ERs, this could negatively impact resilience relative to the other who in less demanding units. Educational background may also be a contributing factor, as the majority of nurses hold bachelor’s degree, which can provide better coping strategies. However, many still face challenges working as bedside nurses under the supervision of directors of nursing, leading to feelings of frustration and stress. Overall, the moderate resilience level reflects a combination of these demographic influences, underscoring the need for targeted interventions to strengthen resilience among nurses, particularly in challenging work environments.

The current findings are in incongruity with a study conducted by Ramirez [44] who revealed greater resilience scores in his study sample, signifying that certain populations may demonstrate higher resilience. Furthermore, Waghmare [45] conveyed moderate levels of staff nurses’ resilience, which are aligned with our findings and reinforced that while nurses have some capacity to cope with stress, there remains significant variability in resilience levels. These results underscore the importance of considering demographic factors and highlight the need for essential interventions to enhance resilience among nursing professionals in various work environments.

Findings from our study highlight the significant negative impact of social undermining on psychological empowerment among nurses, with resilience playing a critical mediating role in alleviating these adverse effects. This underscores the importance of fostering resilience within nursing staff to enhance their empowerment and well-being. Contrariwise, Arshad et al. [46], clarified that resilience may not be sufficient to buffer the more pronounced effects of abusive supervision, indicating that different forms of negative workplace behavior can have varying impacts on employees. Together, these insights emphasize the need for targeted interventions that address both resilience and the specific challenges posed by social undermining and abusive supervision to create healthier work environments and improve overall job satisfaction in nursing.

Among the noteworthy findings of the current study is that, social undermining significantly negatively affects psychological resilience, indicating that higher levels of social undermining led to reduced resilience among nurses. This relationship underscores the harmful impact of negative workplace behaviors on nurses’ ability to cope with stress, potentially resulting in decreased job satisfaction and increased burnout. Addressing social undermining is crucial for fostering a healthier work environment and enhancing nurses’ resilience, ultimately benefiting both nurses and patient outcomes. adversely affects nurses’ resilience by contributing to psychological issues such as loneliness and low self-esteem.

This impairment not only affects their mental health but also their capacity to perform effectively in their roles. On the other hand, a research by Yu et al. [6], signposted that employees with high resilience are better equipped to combat the negative impacts of perceived social undermining. In addition, a recent Spanish study by Zárate-Camargo et al. [47], who analyzed the “Influence of resilience and perceived mistreatment on mental health and the satisfaction with the residency,” indicated that resilience did not correlate with any of the studied variables of concern among medical residents.

Among the crucial findings of the present study is that, there’s a significant positive relationship between psychological empowerment and psychological resilience. In the same track, Aggarwal et al., and Zhai et al.’s recent studies [48, 49] provide robust support for the connection between these constructs. This indicates that individuals with high resilience experience a more pronounced effect of psychological empowerment on their career satisfaction, reinforcing the idea that resilience enhances the benefits derived from empowerment. Collectively, these findings underscore the importance of fostering both psychological empowerment and resilience within the workforce, as they contribute significantly to overall job satisfaction and commitment, ultimately enhancing the quality of the work environment. Also, it highlights the importance of cultivating a culture of empowerment and resilience, which can serve as a powerful tool to encourage registered nurses and midwives to remain in their organizations.

Finally, the findings in Table 4 indicate that the total effect of social undermining on psychological empowerment is both negative and highly significant, underscoring the detrimental impact of such behaviors on individuals’ sense of agency and control within the workplace. This negative association emphasizes how adverse interpersonal dynamics can diminish psychological empowerment, potentially leading to reduced job satisfaction and engagement among employees. Notably, resilience is identified as a critical mediator in this relationship, suggesting that individuals with higher levels of resilience may be better equipped to buffer the adverse effects of social undermining.

The eventual aim of the current work was to explore the role of resilience in the relationship between social undermining and psychological empowerment among nurses. The results verified that psychological resilience mediates social undermining and psychological empowerment. Without the smallest reluctance, this present study is incredible for similarly theoretical and clinical implications, as it clarified the importance and necessity of considering in mind how psychological resilience contribute to a scarcity of detrimental effects of social undermining among nurses, and enhance psychological empowerment in health care institutions, and how imperative it is that nurses should build this capacity.

### Implications for nursing practice

The study’s findings highlight imperious nursing practice considerations for assisting staff nurses in navigating the performance’s obstacles, and enhancing their psychological well-being and professional growth. The study calls for looking into the particular daily behaviors that affect job performance among nurses who experience social undermining either from their supervisors or coworkers engage in that impair their performance. Early social undermining detection should be a top focus for health organizations, and this can be achieved through routine evaluations and preventative strategies as psychological counseling. Furthermore, it is crucial for organizations to seek for promoting ethical leaders through creating an ethical infrastructure, which can forestall social undermining.

Additionally, by fostering resilience, organizations may enhance employees’ ability to maintain their psychological empowerment despite negative workplace interactions. Consequently, these results highlight the importance of implementing interventions aimed at building resilience as a strategy for mitigating the harmful effects of social undermining. The findings call for better recovery programs, strategic human resource leadership practices directed at alleviating factors that exhaust daily self-regulatory psychological resources, and finally top-down interventions preventing burnout among nurses in the healthcare system. Overall, this research underscores the interconnectedness of social undermining and psychological well-being, emphasizing the necessity for supportive organizational environments that promote both resilience and psychological empowerment.

## Conclusion

In deduction, the study’s results are remarkable and applicable to a diversity of nursing scenarios, the existing study’s results revealed that more than half of nurses perceived a high level of overall workplace social undermining. The study finds that social undermining is a workplace stressor that emotionally damages employees by making them feel burnt out when performing their job. It also has a negative influence on psychological empowerment of employees, and in reducing their psychological resilience. The most important finding in our study is that psychological resilience mediates social undermining and psychological empowerment. Finally, the current study may help inspire organizations to pay more attention to the antecedents and consequences of social undermining, directing their efforts on improving or strengthening certain structural and psychological components of the work environments.

## Data Availability

Upon request for scientific purposes, the researcher of correspondence will provide researchable information of the research.

## Abbreviations

PE: Psychological Empowerment
CD-RISC: Connor and Davidson Resilience Scale
PsyES: Psychological Empowerment Scale
SUS: Social Undermining Scale
ICUs: Intensive Care Units
ERs: Emergency Departments
